# Multivariate Calibration Approach for Quantitative Determination of Cell-Line Cross Contamination by Intact Cell Mass Spectrometry and Artificial Neural Networks

**DOI:** 10.1371/journal.pone.0147414

**Published:** 2016-01-28

**Authors:** Elisa Valletta, Lukáš Kučera, Lubomír Prokeš, Filippo Amato, Tiziana Pivetta, Aleš Hampl, Josef Havel, Petr Vaňhara

**Affiliations:** 1 Department of Chemistry, Faculty of Science, Masaryk University, Brno, Czech Republic; 2 Department of Chemical and Geological Sciences, University of Cagliari, Monserrato (CA), Italy; 3 Department of Histology and Embryology, Faculty of Medicine, Masaryk University, Brno, Czech Republic; 4 International Clinical Research Center, St. Anne's University Hospital Brno, Pekarska 53, 656 91, Brno, Czech Republic; Centro de Investigacion y de Estudios Avanzados del Instituto Politecnico Nacional, MEXICO

## Abstract

Cross-contamination of eukaryotic cell lines used in biomedical research represents a highly relevant problem. Analysis of repetitive DNA sequences, such as Short Tandem Repeats (STR), or Simple Sequence Repeats (SSR), is a widely accepted, simple, and commercially available technique to authenticate cell lines. However, it provides only qualitative information that depends on the extent of reference databases for interpretation. In this work, we developed and validated a rapid and routinely applicable method for evaluation of cell culture cross-contamination levels based on mass spectrometric fingerprints of intact mammalian cells coupled with artificial neural networks (ANNs). We used human embryonic stem cells (hESCs) contaminated by either mouse embryonic stem cells (mESCs) or mouse embryonic fibroblasts (MEFs) as a model. We determined the contamination level using a mass spectra database of known calibration mixtures that served as training input for an ANN. The ANN was then capable of correct quantification of the level of contamination of hESCs by mESCs or MEFs. We demonstrate that MS analysis, when linked to proper mathematical instruments, is a tangible tool for unraveling and quantifying heterogeneity in cell cultures. The analysis is applicable in routine scenarios for cell authentication and/or cell phenotyping in general.

## Introduction

In current biomedical research, cells cultured *in vitro* are irreplaceable experimental models and biotechnological tools. However, the research performed on immortalized or tumor-derived cell lines is dependent on proper cell identity and faces continuous significant risk of data misinterpretations due to inadvertent cross-contamination by another cell line [1–4]. Contamination can easily occur by improper passaging, sharing of culture media for multiple cell lines, or inaccurate labeling and storage. Indeed, sophisticated techniques of cell culture and tissue engineering, such as high-throughput reactors, microfluidics, and stem cell or tissue cultures, require stringent monitoring of cell identity and phenotype stability [5–7]. The current gold standard for authentication of individual cell lines is analysis of Short Tandem Repeat sequences (STR) or Simple Sequence Repeats (SSR) widespread throughout the genome, since each cell line theoretically has a unique STR-profile [1,2]. In specific scenarios, such as co-culture setups of two cell lines, use of various cell populations derived from a single individual or from inbred strains, or the occurrence of phenotypic changes within STR- or otherwise stable cell lines, STR analysis cannot provide sufficient discrimination. Furthermore, in cases of intrinsic heterogeneity or impurity of cell cultures, co-culture setups, microsatellite instability, phenotype shifts, or viral or mycoplasma infections, STR analysis can provide ambiguous results, or, in the best case, allow only a qualitative assessment of cell identity without any information on the extent of contamination or heterogeneity in cell populations. Techniques that can complement STR authentication, such as identification of phenotype- or genotype-related markers, e.g. karyotype, isoenzymes, surface markers, or single nucleotide polymorphisms (SNPs) [8,9], are nevertheless dependent on preceding knowledge of the biological background of the model system used. Optimization of high-resolution methods common in physical and analytical chemistry and advanced mathematical modeling can circumvent the need for specific markers by analysis of global cellular or tissue patterns. Spectral techniques, such as Raman near-infrared or mass spectrometry were recently demonstrated to provide global fingerprints with sufficient capacity to distinguish diseased and normal tissues in models of metabolic disorders, or even individual states of cell differentiation or metabolism [10–13].

Matrix-assisted laser desorption/ionization–time of flight mass spectrometry (MALDI-TOF MS) has recently been used in fields beyond analytical and structural chemistry, such as biomedical research or clinical practice, and has been adapted for characterization of complex biological samples by peptide-mass-fingerprinting or peptide sequence tagging. Biotyping of non-fractioned intact microorganisms by MS is now a fast, routine, and cheap technique in clinical microbiology. Similarly, the concept of biotyping eukaryotic cells by intact cell (IC) MALDI-TOF MS has been suggested to allow identification of cell lines [13–18] or to characterize of physiological events occurring in the cells, such as terminal differentiation or programmed cell death [19]. The IC MALDI-TOF MS technique allows for recording the characteristic profiles of eukaryotic cells in quality sufficient for detailed analyses ranging from ultrastructural molecular cytology, to deep cell phenotyping and tissue analyses [13,15,18–22], to species recognition [23] and ecotoxicology [24,25].

However, a simple visual inspection of mass spectra is often not sufficient to establish an unambiguous cell line-specific set of biomarker peaks. Moreover, specific signal intensity and analytic concentration are not linear except in a narrow concentration range due to various stochastic “MALDI effects,” such as variability in matrix/analyte interactions and energy dissipation or quenching or enhancing of ionization [26]. Mathematical methods that are successfully used in chemometrics, such as bivariate regression, polynomial fitting, multiple linear regression, partial least squares, and artificial intelligence, must therefore be applied in MS analysis of complex biological samples [27–29]. Artificial neural networks (ANNs) represent a robust and versatile mathematical tool for many applications in various fields [30]. ANNs mimic the “*learning*” and “*generalization*” abilities of human neural structures. ANNs are able to model highly complex non-linear systems and are used for classification, pattern recognition, modeling, and multivariate data analysis [31]. The basic units of ANNs are “*nodes*” or “*neurons*.” They are organized in one “*input”* layer, in one or more “*hidden”* layers, and in one “*output”* layer. Each of the *i*-th neurons in a layer is linked to all the *j*-th neurons in the next layer. Each connection is weighted with a weight *w*_*ij*_. The role of the neurons in the input layer is to receive input data and transfer it to the neurons in the hidden layer through the weighted connections. The neurons in the hidden layer(s) perform mathematical operations on the incoming data (summation, addition of a “*bias*” term, and transformation by a suitable mathematical function). The result is then transferred to the neurons in the output layer where the ANN output is calculated.

Here we study the possibility of using ANNs to determine a quantity of cells of a particular cell line and/or type in two-component mixtures, mimicking a scenario of cell line cross-contamination. To create such situations, we used: a) line CCTL14 of human embryonic stem cells (hESCs), b) mouse embryonic fibroblasts (MEFs), and c) line R1 of mouse embryonic stem cell (mESCs). The cells were arranged into two-component calibration mixtures of hESCs + MEFs and hESCs + mESCs in various ratios. Line CCTL14 of hESCs has previously been thoroughly characterized [32]. MEFs freshly isolated from connective tissue of 11.5 days old mouse embryos are commonly used as a supportive feeder layer for hESCs in a routine co-culture mode. Mouse ESCs [33] represent pluripotent and self-renewing cells that are developmentally and functionally similar to hESCs.

The two-component cell suspensions were analyzed by ANN-coupled IC MALDI-TOF MS in a multivariate calibration approach. We demonstrate that mass spectra contain sufficient information to identify the presence of individual cell types in mixtures, and we report for the first time that ANN analysis of mass spectra from two-component mixtures can correctly predict the level of cell cross-contamination in very complex microenvironment.

## Material and Methods

### Chemicals

Knockout Dulbecco’s modified Eagle’s medium (DMEM), DMEM/F12, knockout serum replacement, fetal bovine serum, L-glutamine, minimum essential medium non-essential amino acids, penicillin-streptomycin, and TrypLE^TM^ Select were purchased from Gibco, Life Technologies Czech Republic Ltd. (Prague, Czech Republic). Ammonium bicarbonate, sinapinic acid, trifluoroacetic acid, DMEM, 2-mercaptoethanol, phosphate buffered saline (PBS), and gelatin were purchased from Sigma-Aldrich Ltd. (Prague, Czech Republic). Matrigel^TM^ was purchased from BD Bioscience, I.T.A.-Intertact Ltd. (Prague, Czech Republic). Fibroblast growth factor-2 was purchased from PeproTech, Baria Ltd. (Prague, Czech Republic). Leukemia Inhibitory Factor Protein was purchased from Chemicon, Merck Millipore (Prague, Czech Republic). Acetonitrile was purchased from J.T. Baker, VWR International Ltd. (Prague, Czech Republic). PepMix standard was purchased from Laser BioLabs (Sophia-Antipolis, France). Tissue culture dishes were purchased from TPP (Trasadingen, Switzerland).

### Cell cultures

Mouse embryonic fibroblasts derived from CF1-mouse embryos were cultured in tissue culture dishes in medium consisting of Knockout DMEM supplemented with 10% fetal bovine serum, 2 mM L-glutamine, 1% minimum essential medium non-essential amino acids, 1% penicillin-streptomycin, and 0.1 mM 2-mercaptoethanol as described previously [34,35]. Human embryonic stem cells [36,37] were cultured in the undifferentiated state in tissue culture dishes coated with Matrigel^TM^ in culture media conditioned by MEFs consisting of DMEM/F12 supplemented with 15% knockout serum replacement, 2 mM L-glutamine, 1% minimum essential medium non-essential amino acids, 0.5% penicillin-streptomycin, 0.1 mM 2-mercaptoethanol, and 4 ng/ml fibroblast growth factor-2. Mouse embryonic stem cells [33] were cultured in tissue culture dishes coated with 0.1% gelatin in medium consisting of DMEM supplemented with 20% fetal bovine serum, 1% minimum essential medium non-essential amino acids, 1% penicillin-streptomycin, 1 mM 2-mercaptoethanol and 5.5 μg/ml leukemia inhibitory factor (LIF). All cell lines were maintained in an incubator at 37°C with a humidified atmosphere containing 5% CO_2_, with daily media exchange.

### Sample preparation

Cultured cells were washed with 1×PBS and enzymatically disaggregated to single cell suspension using TrypLE™ Select. After 2 min, the enzymatic activity was stopped by the respective culture medium. Detached cells were pelleted by centrifugation at 200 g for 5 min and washed once again with 1×PBS. Cell number was determined by CEDEX XS cell counter operated with CEDEX Control Center software v. 1.0.3. from Innovatis AG, Roche Life Sciences (Prague, Czech Republic).

### Preparation of cell mixtures for MS analysis

#### hESCs + MEFs mixture

Cell suspensions containing a total of 1×10^6^ hESCs and MEFs in 1×PBS in defined ratios were pelleted by centrifugation at 200 g for 5 min at 4°C and washed three times with an aqueous solution of 150 mM ammonium bicarbonate. Then, the cell pellets were resuspended in 10 μl of 150 mM ammonium bicarbonate and mixed with 5 μl of freshly prepared sinapinic acid matrix (30 mg/ml in 70% acetonitrile and 7.5% trifluoroacetic acid). Two microliters of sample/matrix mixture were immediately spotted in pentaplicates onto the MALDI target and dried at room temperature.

#### hESCs + mESCs mixture

Aliquots of 1×10^6^ mESCs or hESCs in 1×PBS were pelleted by centrifugation at 200 g for 5 min at 4°C. Supernatant was discarded and pelleted cells were washed three times with 150 mM ammonium bicarbonate solution. Resulting cell pellets were then snap-frozen and stored until further processing. At the time of analysis, both mESCs and hESCs aliquots were quickly thawed and reconstituted in 20 μl of 150 mM ammonium bicarbonate solution and sonicated briefly in a water ultrasound bath. Then, MEFs and hESCs were mixed in given ratios to a total of 0.5×10^6^ cells per sample and total volume was adjusted to 15 μl with 150 mM ammonium bicarbonate. Each cell suspension was mixed with 7.5 μl of freshly prepared sinapinic acid matrix solution. Two microliters of sample/matrix mixture were immediately spotted onto the MALDI target and dried at room temperature. Each sample was spotted in five technical replicates.

### Mass spectrometry

Mass spectra were recorded on an AXIMA CFR mass spectrometer from Kratos Analytical (Manchester, UK) in linear positive ion mode. The instrument was equipped with a nitrogen laser (337 nm) and delayed extraction was used. The laser energy was expressed in arbitrary units from 0 to 180 a.u. The power of the laser at 180 a.u. was 6 mW, while the irradiated spot size was approximately 150 μm in diameter. External mass calibration was done using the PepMix4 standard. The laser repetition rate was 5 Hz with a pulse time width of 3 ns. Each mass spectrum was obtained by the accumulation of at least 5000 shots. In order to decrease the contribution of chemical noise and possible errors on the baseline and in calibration, the raw mass spectra were pre-processed, cleaned, transformed, and reduced in dimensionality before the data analysis, as described elsewhere [38].

Mass spectra were analyzed using Launchpad Software (Kompact version 2.9.3, 2011) from Kratos Analytical Ltd. Pre-processing of mass spectra and ANN computation were performed using MATLAB 8.6. 2015 from The MathWorks Inc. (Natick, Massachusetts, USA) and Trajan Neural Network Simulator, Release 3.0 D 1996–1998, from Trajan Software Ltd. (Durham, U.K.). Partial least squares projection to latent structures regression was performed with “leave-one-out” cross-validated prediction in program R (www.r-project.org) using the external *pls* library [39].

### Artificial neural networks

We constructed an artificial neural network containing four neurons in one hidden layer. The intensities of processed mass spectra served as the input, while the number of contaminating cells in the two-component mixtures was the output. The “*learning*” of the ANN was performed using the back-propagation training algorithm as described elsewhere [40,41]. The back-propagation was achieved by iteratively adjusting the values of connection weights in order to minimize the difference between the ANN calculated output value (o*_pk_) and the experimental one (*o*_*pk*_). After each iteration, the root mean square of the sum of (*o*_*pk*_ -o*_pk_)^2^ residuals (RMS) was calculated according to Eq 1:

$$\text{RMS}=\;\sqrt{\frac{\sum_{p=1}^{N}\sum_{k=1}^{M}{\left(o_{pk}-o_{pk}^{*}\right)}^{2}}{N\times M}}$$ (1)

where *N* is the number of mass spectra, *M* is the number of outputs, o*_pk_ is the ANN calculated, and *o*_*pk*_ is the experimental output value. The optimal ANN architecture was confirmed by plotting the RMS value against the number of neurons in the hidden layer(s) and number of training cycles (epochs).

## Results And Discussion

### Preparation of two-component mixtures of different cell types and intact cell MALDI-TOF mass spectrometry

**Fig 1:**
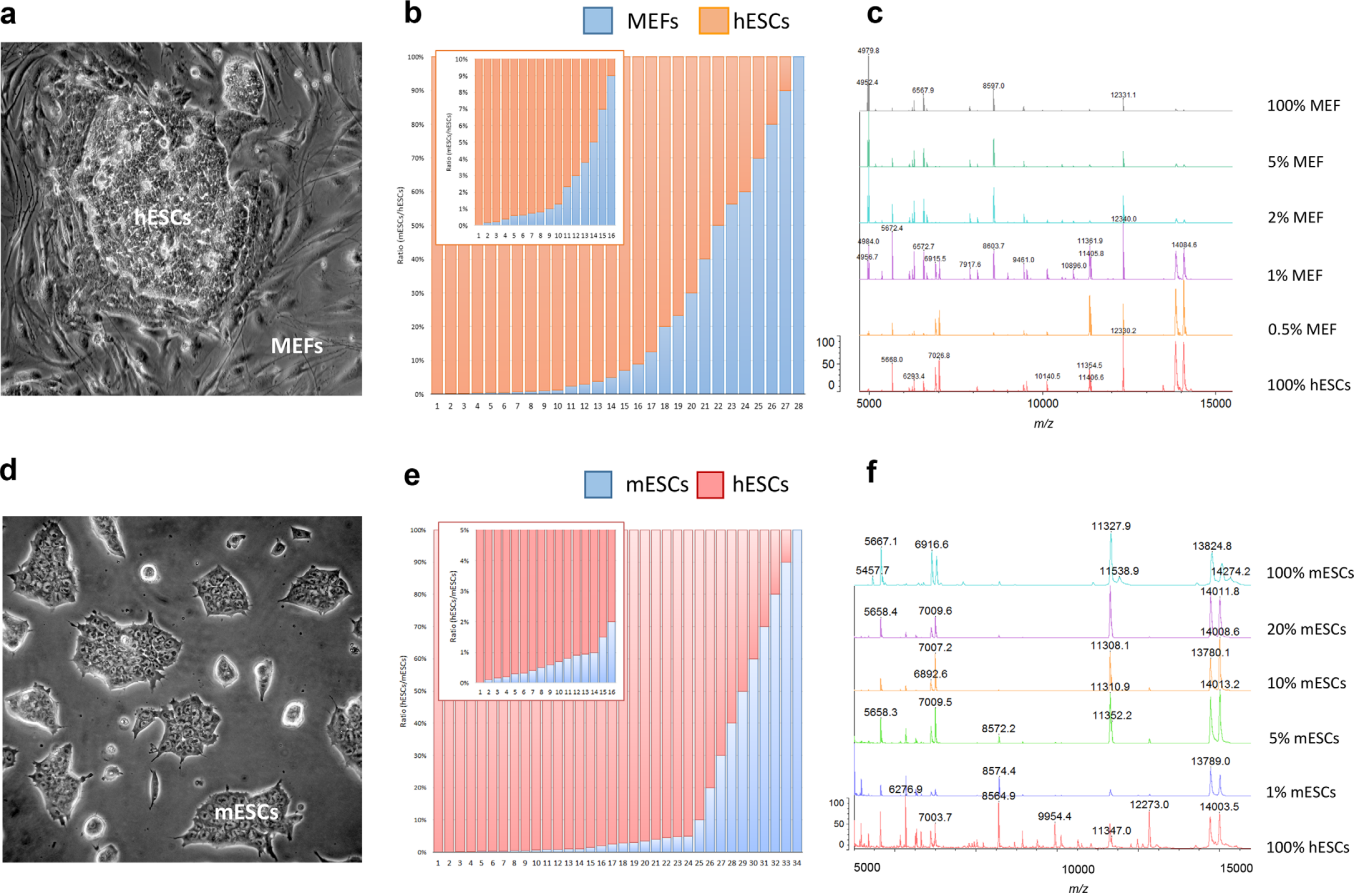
(A) Colony of human embryonic stem cells (hESCs) cultured on a feeder layer of mouse embryonic fibroblasts. (B) Experimental ratios of two-component mixtures of hESCs + MEFs. (C) Representative mass spectra of selected two-component mixtures of hESCs + MEFs. (D) Colony of mouse embryonic stem cells (mESCs) in a feeder-free culture. (E) Experimental ratios of two-component mixtures of hESCs + mESCs. (F) Representative mass spectra of selected two-component mixtures of hESCs + mESCs.

We prepared calibration datasets consisting of twenty-eight defined two-component mixtures of hESCs + MEFs, thirty-four mixtures of hESCs + mESCs (**Fig 1A and 1D)**, and pure cell populations, with total cell numbers of 1×10^6^ (**Fig 1B and 1E**). We then recorded the mass spectra of two-component cell mixtures and pure cell populations in the 2000–20000 m/z range without previous fractionation or extraction. We pre-processed the mass spectra by *(i)* resampling to 30000 m/z values (homogenizing in a chosen range and reducing the number of m/z values), *(ii)* aligning (removing the systematic shifts in mass spectra of repeated experiments), *(iii)* baseline subtraction, *(iv)* smoothing, and *(v)* normalization to a vector of unit length **(Σ*X***_***i***_ **= 1**), where *X*_*i*_ are the intensities of the peaks of the mass spectrum) [42] (**Fig 1C and 1F, S1 Fig**). Next, we organized the spectral data into a matrix with dimensions *m* × *n*, where *m* represents the number of the mass spectrum of the particular cell mixture and *n* are the m/z values. The *i-th* row of the matrix represents the mass spectrometric fingerprint of the *i-th* mixture. In order to decrease the latent noise in pre-processed mass spectra, we selected only the peaks with intensity higher than an arbitrarily set threshold (1×10^−3^) for further analysis. Thus, the data matrices of hESCs + MEFs and hESCs + mESCs were reduced from the original *n* × 30000 to *n* × 84 and *n* × 122, respectively. Next, we identified peaks with the highest intrinsic variability in the datasets, as described elsewhere [43–46]. In brief, we selected informative peaks by comparative determination of standard deviations of individual peaks normalized to the total variance of the dataset, Lasso regression, and sparse partial least squares regression. The informative peaks are visualized in **S3 Fig**. That allowed us to finally reduce the data matrices of hESCs + MEFs and hESCs + mESCs to *n* × 10 and *n* × 30, respectively (**S1 Table**).

**Fig 2:**
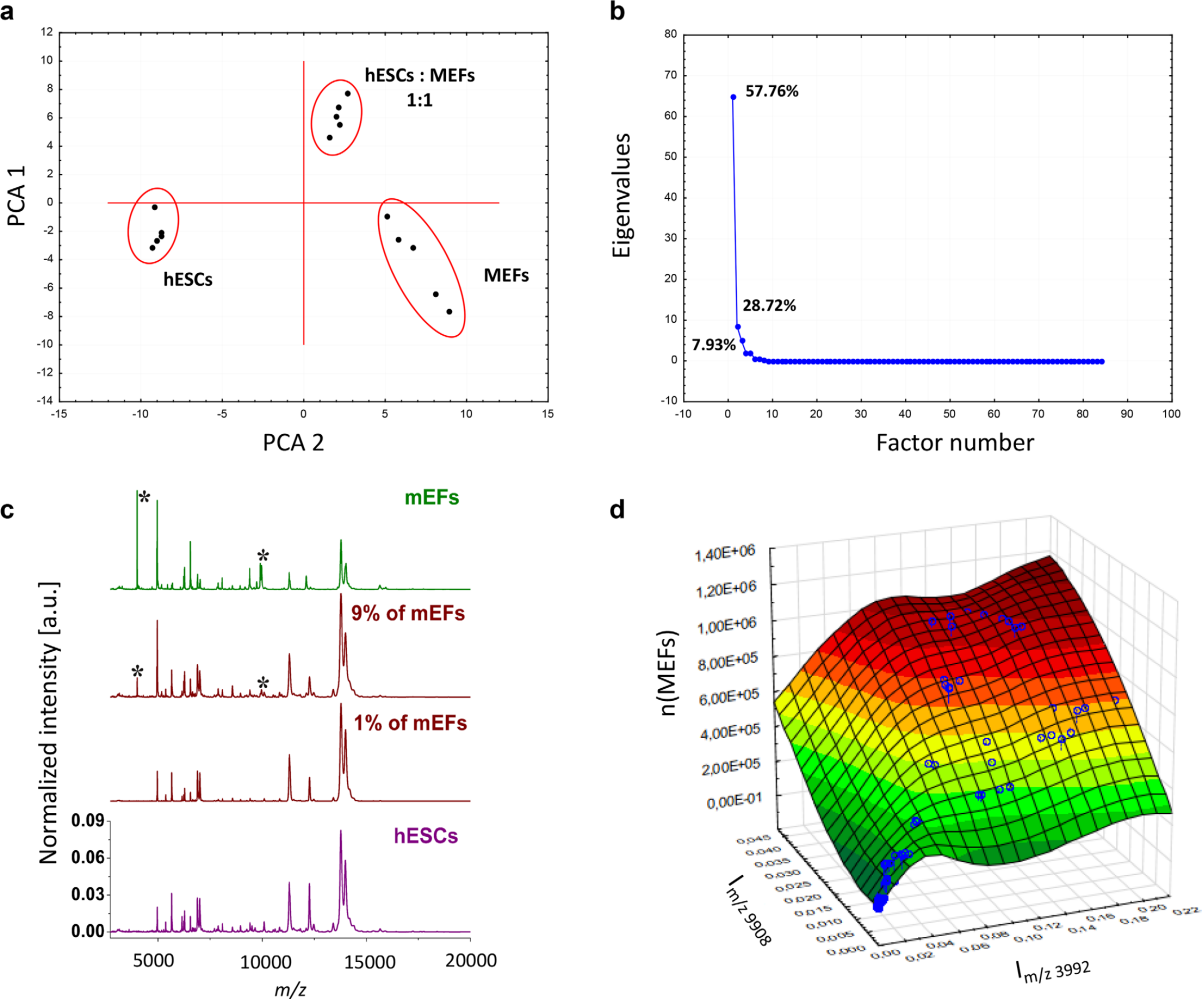
(A) Principal component analysis of mass spectra dataset containing intensities of 84 m/z for pure MEF and hESC populations and their 1:1 mixture. (B) Scree plot documenting the presence of three factors contributing predominantly to the overall variability in the analyzed dataset. (C) Pre-processed MALDI-TOF mass spectra for pure hESCs and MEFs and a hESCs + MEFs two-component mixture containing 99% hESCs and 1% MEFs. The spectra were normalized to vector of unit length (a.u.). Asterisks indicate peaks at m/z 3992 and 9908. (D) Surface plot of intensities of peaks at m/z 3992 and 9908 versus the number of MEFs in hESCs + MEFs two-component mixtures.

Then, we used these datasets to perform principal component analysis aiming to discriminate the pure hESC and MEF cell populations and the cell mixtures containing 50% of each cell type. Eigenvalue analysis showed the presence of three factors contributing up to 94% of the overall variability. Plotting the principal components revealed three clearly separated clusters and provided proof of principle for the discriminative information of the MEF and hESC mass spectra (**Fig 2A and 2B**). Similar discrimination was achieved for mESCs and hESCs (data not shown).

Next, we visually compared mass spectra obtained from the pure hESCs and MEFs, and their two-component mixtures. Despite the high similarity of the mass spectra, we identified peaks unique to hESCs and MEFs (m/z 3992 and 9908) (**Fig 2C**) appearing reproducibly over various mixtures. We presumed that if these two marker peaks are informative for MEFs, their intensities should be proportional to the content of MEFs in the two-component mixtures. However, we did not identify any linear trend between the normalized intensities of these two marker peaks and the percentage of MEFs, especially in two-component mixtures with low concentrations of MEFs (**Fig 2D**). In the case of highly similar pluripotent cell types, the mass spectra of hESCs + mESCs mixtures lacked any spectral patterns specific for individual pure cell lines (**Fig 1F**). Therefore, assessment of the individual biomarker peaks was not suitable for precise and unambiguous quantification.

### Quantitative determination of contamination levels

Because of the data complexity, it was difficult to handle the mass spectral datasets by simple linear analyses. We first examined the data by a method of partial least squares with projection to latent structures regression (PLS) on the full data matrix of the complete mass spectra. PLS has been developed and extended by Herman and Svante Wold, respectively, [47] for quantitative analysis of highly complex multivariate data and is used preferentially in chemometrics. Despite a correlation between predicted and actual cell percentages in the two-component mixtures, the prediction precision by PLS was rather low, with substantial root mean square error (RMS) showing signs of systematic trends (**Table 1, S3 Fig**). Therefore, we asked whether non-linear approaches and artificial intelligence methods, such as ANNs, could make predictions with more precision. ANNs were previously reported to provide effective analysis and classification of biological, clinical or bioanalytical, and chemometric non-linear data (for review see [30]), and were found particularly suitable for analysis of MS data [28].

**Table 1:** Values of RMS calculated as differences between predicted and observed values, *k* (regression coefficients), and *R*^*2*^ (determination coefficient).

	hESCs: MEFs		hESCs: mESCs	
	PLS	ANN	PLS	ANN
**RMS**	51.7×10^3^	3.16×10^3^	96.6×10^3^	7.1×10^3^
**k**	0.976	0.996	0.601	0.975
**R**^**2**^	0.9759	0.9992	0.6010	0.9822

**Fig 3:**
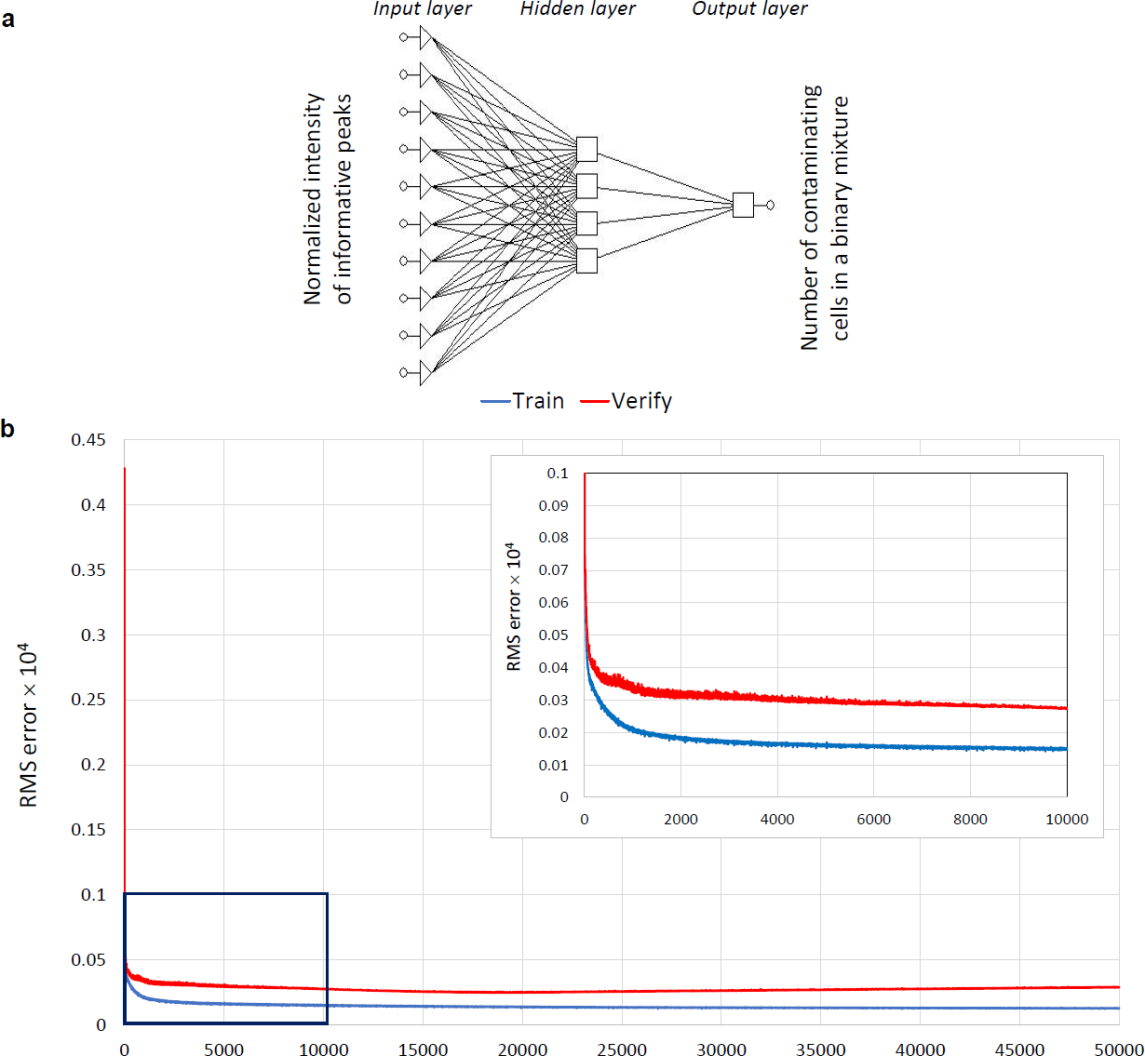
(A) Optimal ANN architecture (one Input layer, one Hidden layer with four neurons, and one Output layer). (B) Training and leave-one-out verification plot of the RMS versus the number of training cycles (epochs). First 50 000 iterations are shown. The inset shows a detailed plot for the first 10 000 training cycles.

**Fig 4:**
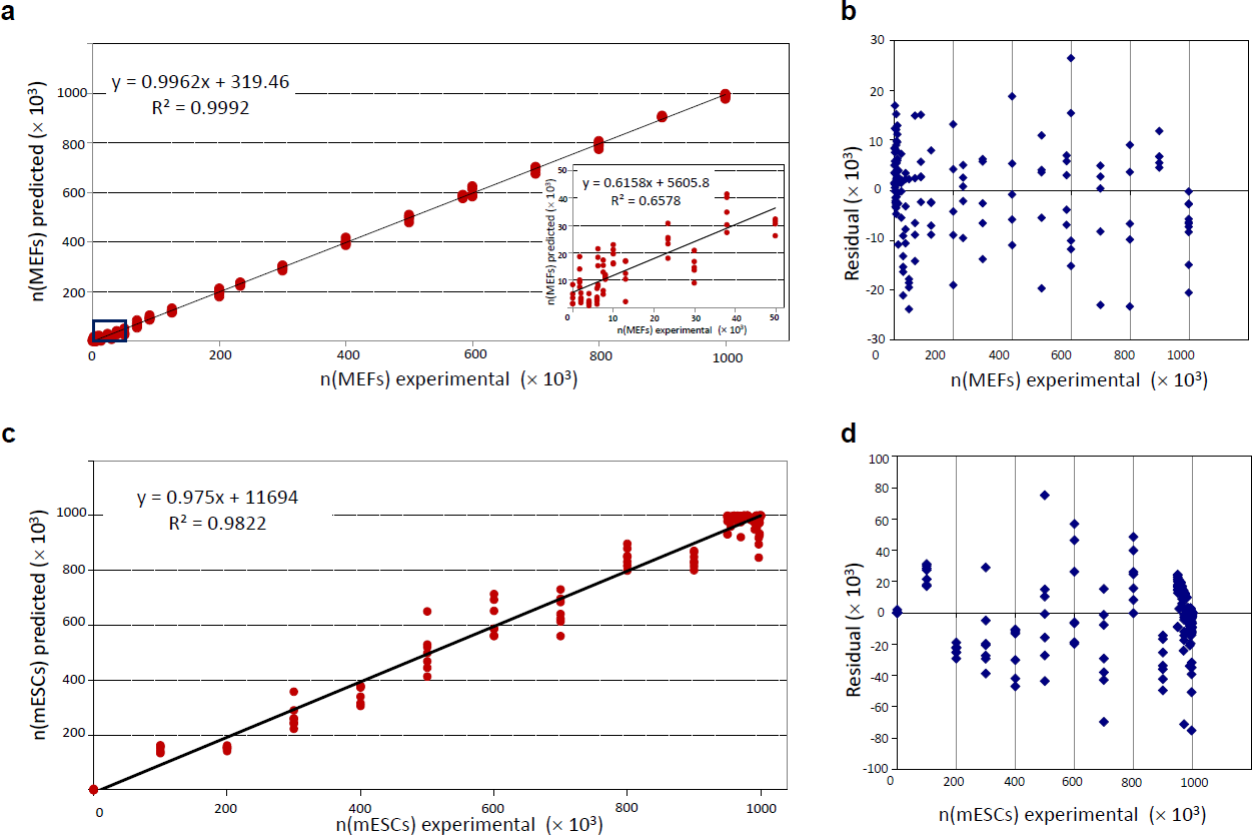
(A) Correlation between ANN-predicted number of cells and the experimental number of MEFs in two-component mixtures of hESCs + MEFs. The inset shows the correlation between experimental and predicted values in low concentration ranges of MEFs up to the 50×10^3^ cells in the two-component mixtures. (B) Overview of Residuals (difference between ANN-predicted number of cells and the experimental values) versus the experimental number of MEF cells in two-component mixtures of hESCs + MEFs. (C) Correlation between ANN-predicted number of cells and the experimental number of mESCs in two-component mixtures of hESCs + mESCs. (D) Overview of Residuals (difference between ANN-predicted number of cells and the experimental values) versus the experimental number of mESC cells in the two-component mixtures of hESCs + mESCs.

In our analysis the intensities of the selected peaks comprised the ANN-input data and the number of MEFs and/or mESCs cells in the hESC calibration mixtures was the ANN-output data. For the training step of the ANN, we tested several algorithms and found the back-propagation algorithm to be the most suitable (data not shown). We determined the optimal architecture containing four neurons in one hidden layer (**Fig 3A**) by plotting the RMS against the number of nodes (data not shown) and we validated over 100000 training cycles (epochs), without overfitting the model (**Fig 3B**). We used the leave-one-out cross-validation method to test the “*generalization*” ability of the designed network to predict the single cases excluded from the training data set. We used the RMS as a measure of the prediction accuracy. The network was able to evaluate the input data and correctly predict the number of MEFs (**Fig 4A**) and mESCs (**Fig 4C**) in the hESC suspensions over the whole range of evaluated ratios. Prediction by ANN was correct even at low percentages of contaminating cells in suspension. To validate the model, the RMS was calculated as the differences between the predicted and experimental values. The residuals reached significantly lower values than in PLS predictions (**Table 1**). Moreover, the residual values were randomly distributed, and the absence of any systematic error or trend in residuals demonstrated correctness of the model (**Fig 4B and 4D**). To perform further validation of the method, we analyzed an independent dataset of fifty hESCs + MEFs mixtures. Using a training set described above, the ANN correctly determined the numbers of MEFs in hESCs suspension with high correlation between predicted and experimental values **(S4 Fig)**. In summary, multivariate calibration coupled with a correctly trained ANN was able to determine the ratio of cell numbers in two-component mixtures.

We have identified the conditions and developed a step-by-step protocol for successful quantitation of two distinct cell types in a single two-component mixture by a multivariate calibration approach based on an ANN-coupled IC MALDI-TOF MS analysis. The major steps of the method include:

1. *a priori* knowledge or identification of the contaminating cell line
2. construction of two-component calibration mixtures of the given cell lines
3. mass spectra pre-processing and m/z selection
4. parallel recording of mass spectra of pure cell populations and calibration mixtures and building a library of spectral datasets for multivariate calibration
5. estimation of the contamination levels in unknown samples using an ANN model trained on the calibration datasets

**Fig 5:**
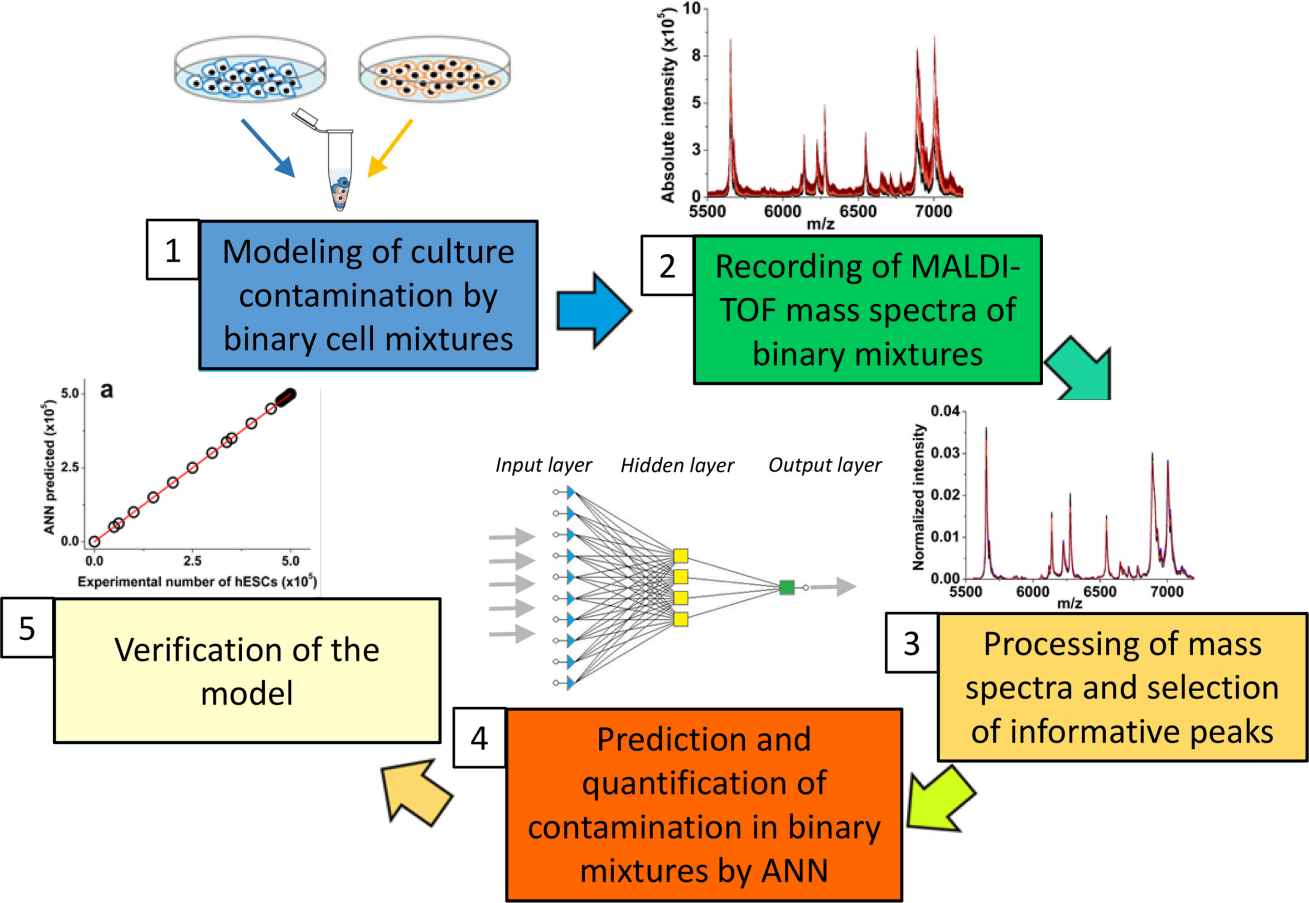
Overview for quantitative ANN-coupled MS-based analysis of cross-contamination.

**Fig 6:**
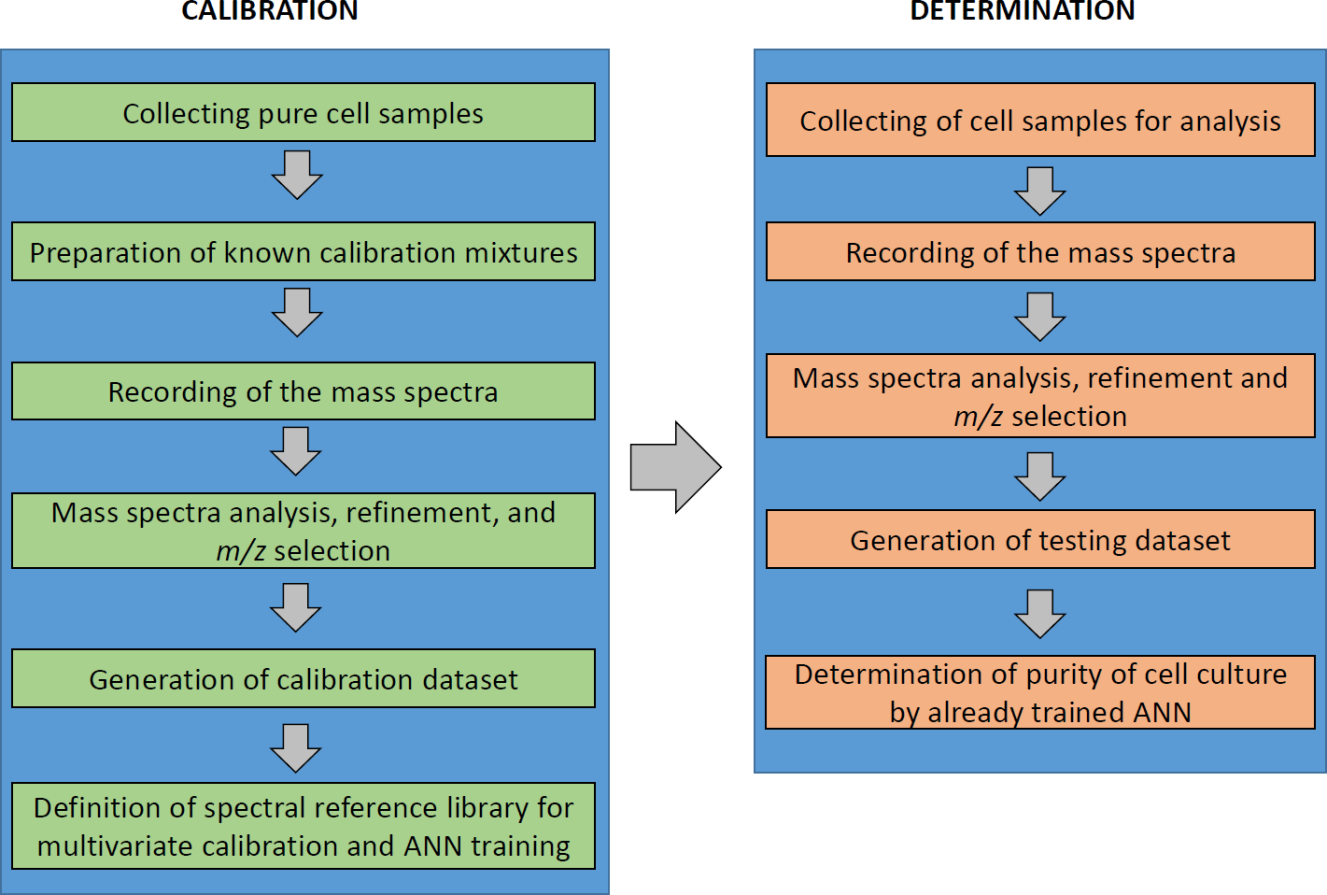
Experimental schematic of the multivariate calibration-based ANN spectral analysis.

The application of ANNs allowed us to overcome the unwanted inconsistency and non-linearity of IC MALDI-TOF MS spectra and reveal hidden patterns in mass spectra to unambiguously identify and quantify MEFs or mESCs in the hESC culture. However, *a priori* knowledge of the contaminating cell line is a prerequisite for correct prediction and selection of the training dataset. The multivariate calibration-based ANN approach can be easily adapted to routine protocols for quantitative determination of cell culture homogeneity and consistency and for thorough MS analyses of cell parameters in various culture platforms, with all steps adaptable for any experimental, routine, or high-throughput culture setup [48,49] (**Figs 5 and 6**). Currently, methods involving assessment of cell quality in clinical grade cultures, biomedical research or bio-industry involve either genetic authentication confirming the cell identity or functional assays documenting the phenotype. The intact cell mass spectrometry coupled with ANN can reveal inconsistencies occurring in high-throughput or long-term cultures or co-cultures, by monitoring spectral patterns and their alterations.

In conclusion, we demonstrated for the first time that the multivariate calibration approach based on ANN-coupled IC MALDI-TOF MS analysis can provide quantitative information on cell culture heterogeneity and authenticity and thus complement the portfolio of techniques that are available for characterization of mammalian cell cultures.

## Supporting Information

S1 Fig Comparison of raw (a) and pre-processed (b) mass spectra (five replicates) characterizing human embryonic stem cells (hESCs). The pre-processed mass spectra were normalized to a vector of unit length (Σ*X*_*i*_ = 1), where *X*_*i*_ are the intensities of the peaks of the mass spectrum).
(TIF)

S2 Fig Standard deviations of the mean intensity of peaks of the particular m/z in the dataset normalized to the standard deviation of the mean intensity of the hESCs + MEFs (a) and hESCs + mESCs dataset (b). Peaks used in the further analyses are indicated by respective m/z values.
(TIF)

S3 Fig Correlation between PLS-predicted number of cells and the experimental number of MEFs in two-component mixtures of (a) hESCs + MEFs and (b) hESCs + mESCs. Overview of Residuals (difference between PLS-predicted number of cells and the experimental values) versus the experimental number of cells in the two-component mixtures of (c) hESCs + MEFs and (d) hESCs + mESCs. (TIF)

S4 Fig Training and verification plot for an independent dataset of 50 cases of hESCs + MEFs mixtures (a). Correlation between experimental and ANN-predicted numbers of MEFs in hESCs suspension.
(TIF)

S1 Table Overview of normalized mass-to-charge ratios (m/z) of informative peaks used for analysis. (TIF)
