# Supplementary figures and images for: Multivariate Calibration Approach for Quantitative Determination of Cell-Line Cross Contamination by Intact Cell Mass Spectrometry and Artificial Neural Networks
Source: PLoS One. 2016 Jan 28;11(1):e0147414. doi: 10.1371/journal.pone.0147414 (PMC4731057; doi:10.1371/journal.pone.0147414)

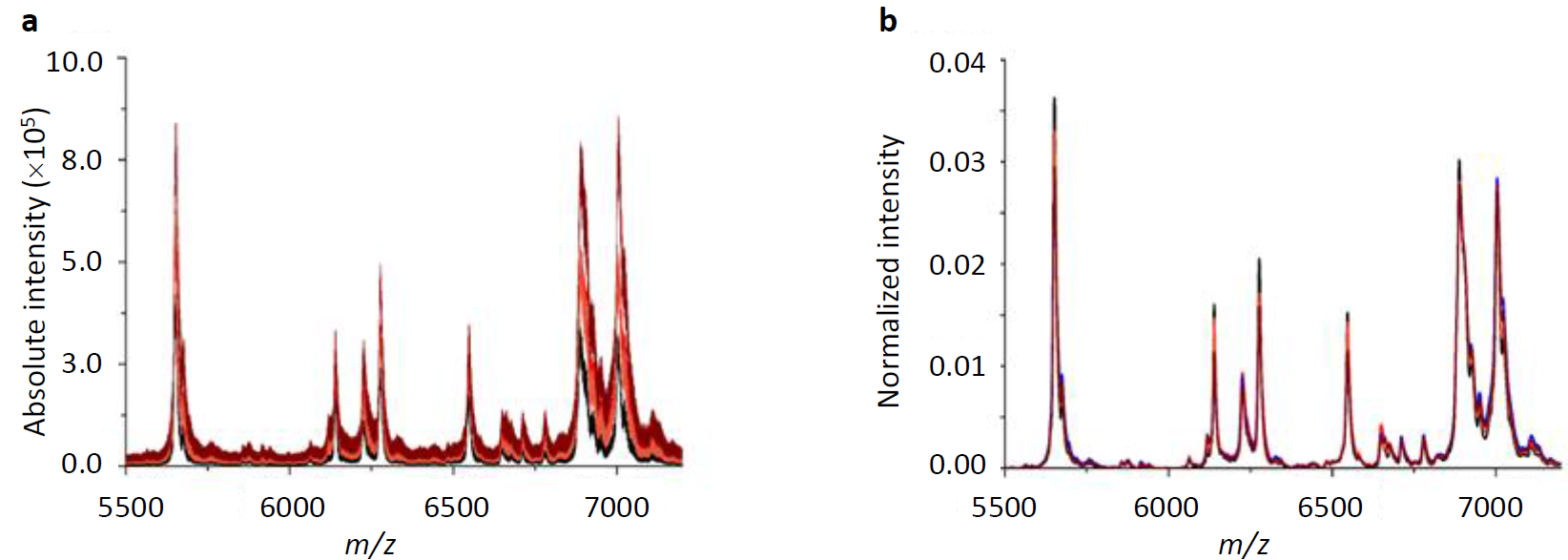

Supplement: S1 Fig — The pre-processed mass spectra were normalized to a vector of unit length (ΣXi = 1), where Xi are the intensities of the peaks of the mass spectrum). (TIF) [file pone.0147414.s001.tif]

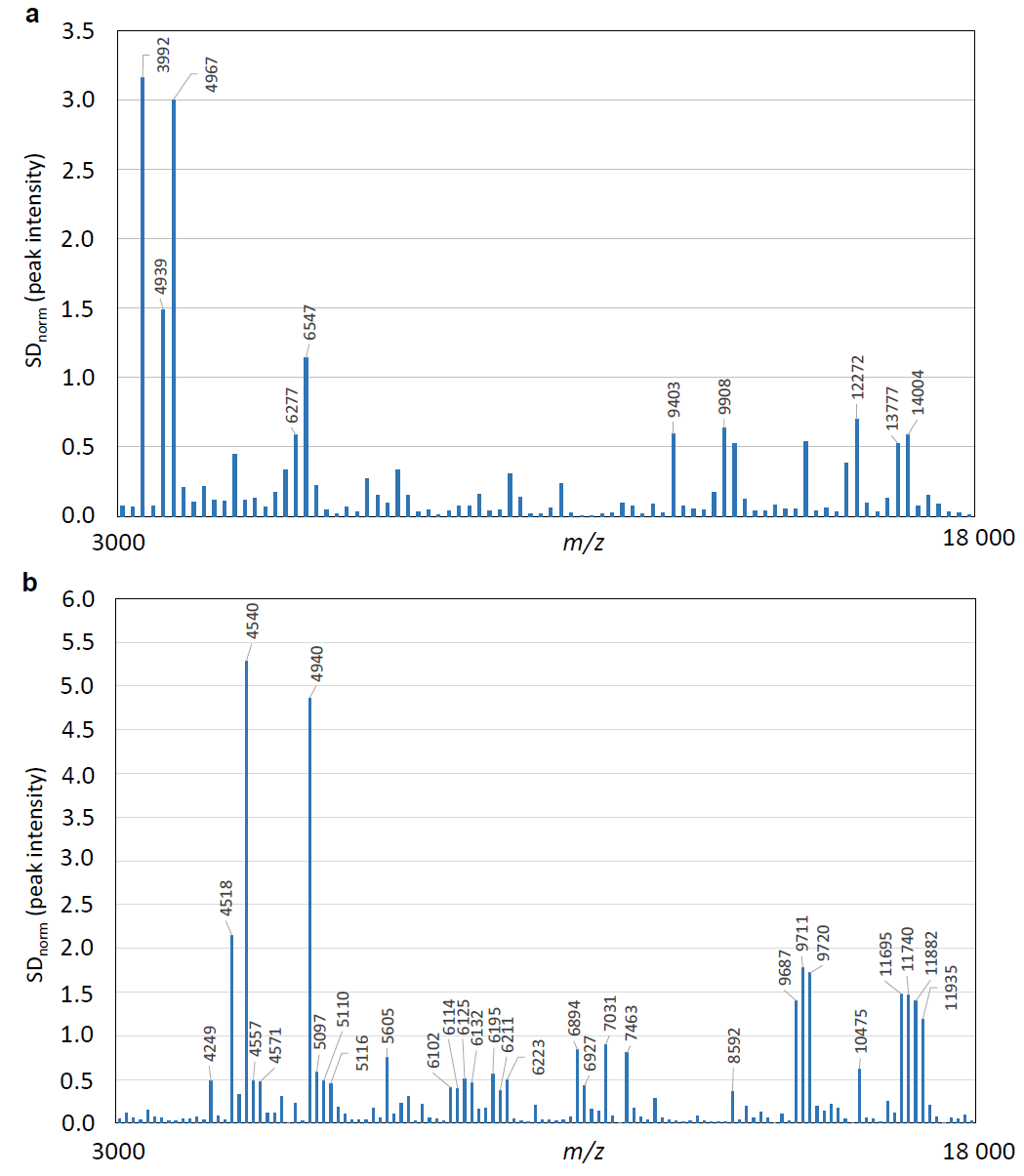

Supplement: S2 Fig — Peaks used in the further analyses are indicated by respective m/z values. (TIF) [file pone.0147414.s002.tif]

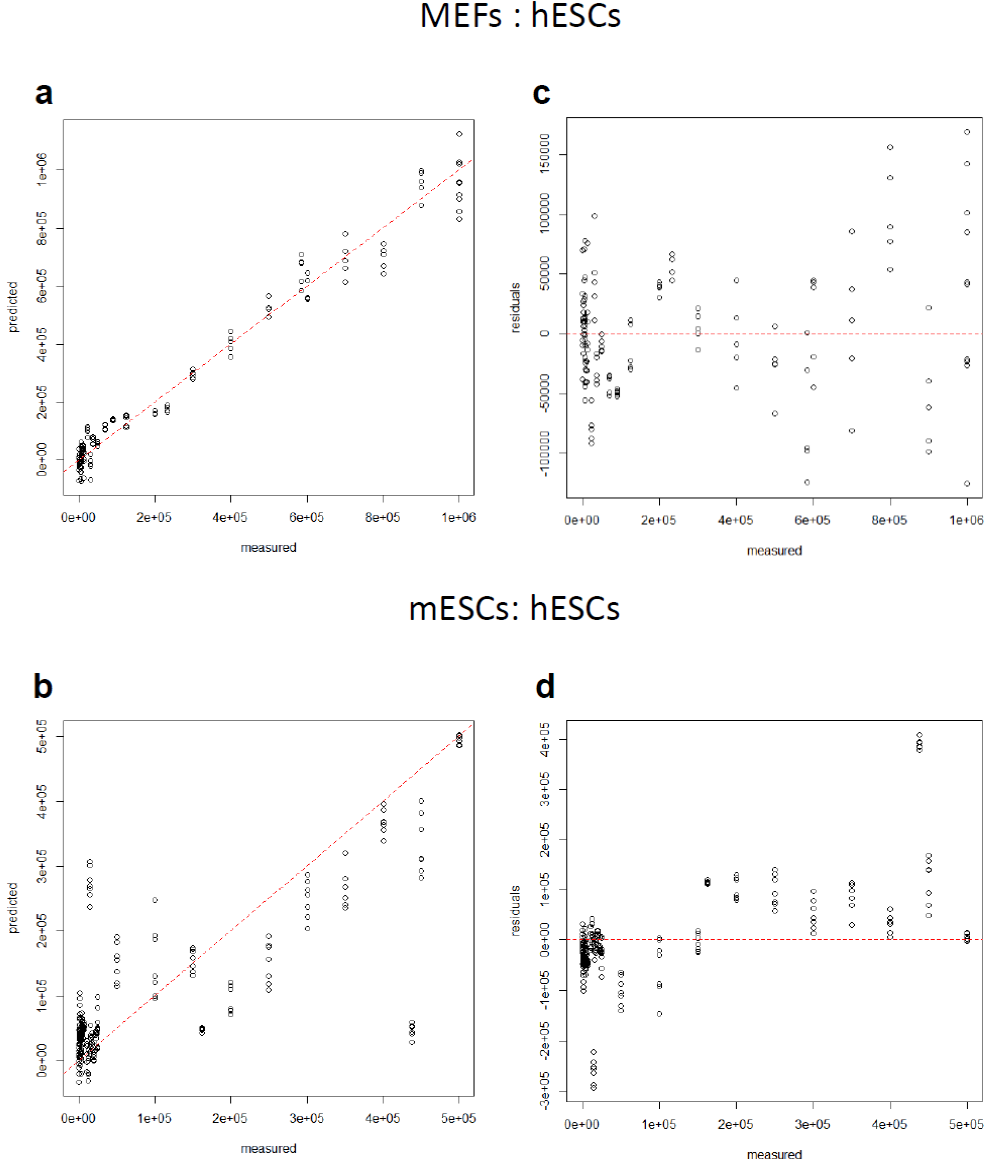

Supplement: S3 Fig — (TIF) [file pone.0147414.s003.tif]

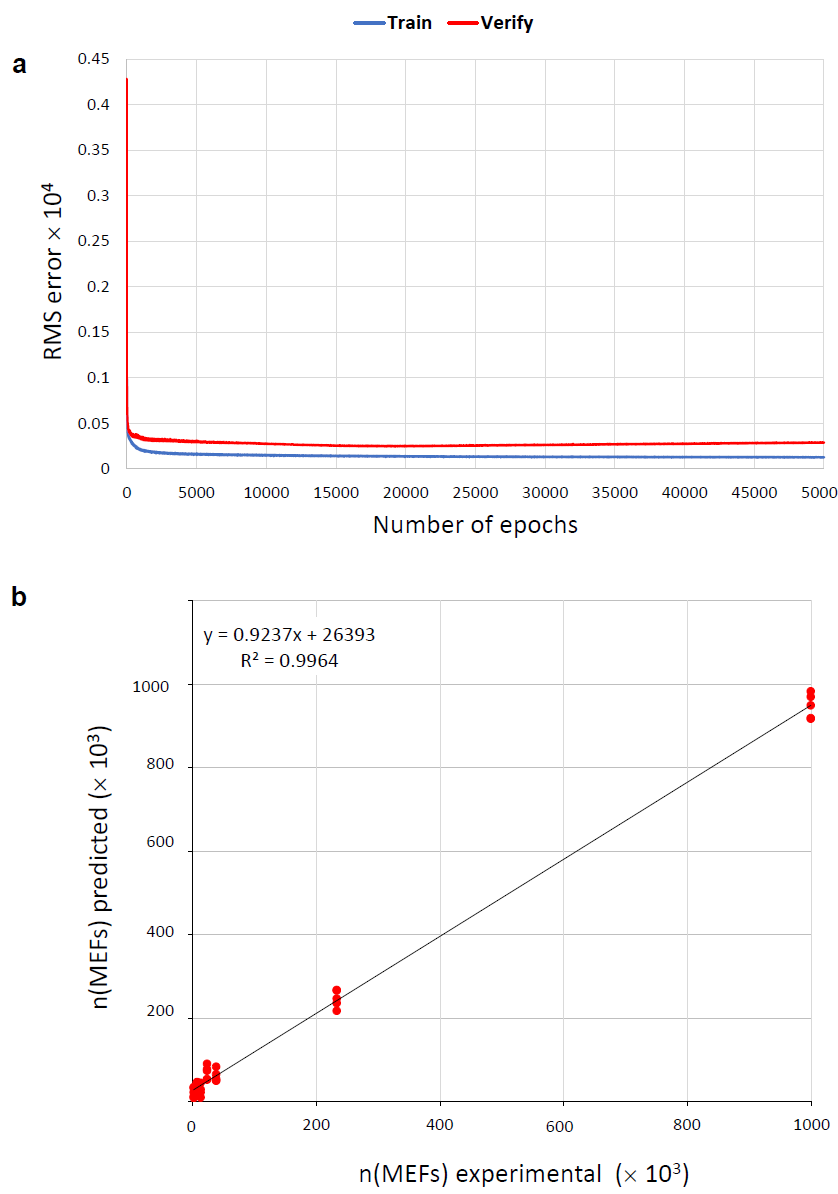

Supplement: S4 Fig — Correlation between experimental and ANN-predicted numbers of MEFs in hESCs suspension. (TIF) [file pone.0147414.s004.tif]

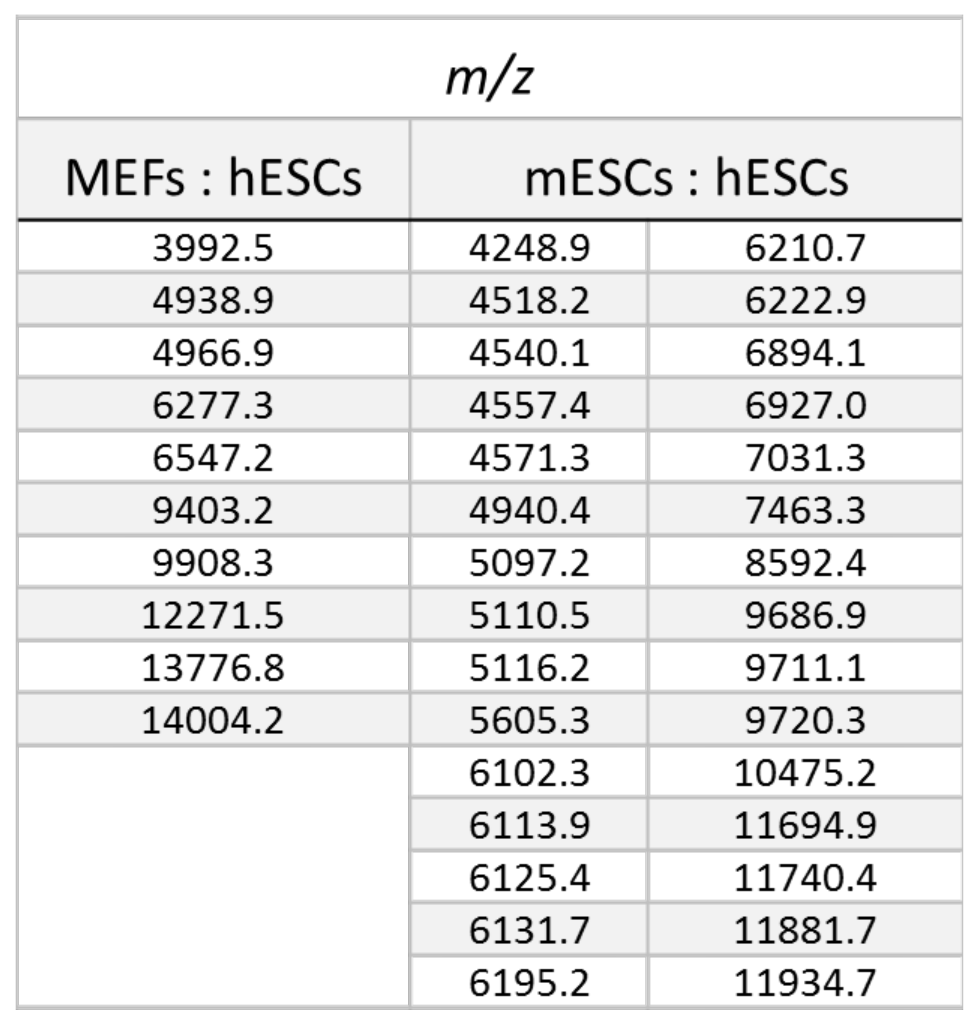

Supplement: S1 Table — (TIF) [file pone.0147414.s005.tif]
